# Preoperative Age and Its Impact on Long-Term Renal Functional Decline after Robotic-Assisted Partial Nephrectomy: Insights from a Tertiary Referral Center

**DOI:** 10.3390/medicina60030463

**Published:** 2024-03-11

**Authors:** Cesare Saitta, Giuseppe Garofano, Giovanni Lughezzani, Margaret F. Meagher, Kit L. Yuen, Vittorio Fasulo, Pietro Diana, Alessandro Uleri, Andrea Piccolini, Stefano Mancon, Paola Arena, Federica Sordelli, Matilde Mantovani, Pier Paolo Avolio, Edoardo Beatrici, Rodolfo F. Hurle, Massimo Lazzeri, Alberto Saita, Paolo Casale, Ithaar H. Derweesh, Marco Paciotti, Nicolò M. Buffi

**Affiliations:** 1 Department of Biomedical Sciences, Humanitas University, 20072 Pieve Emanuele, Italy; cesare.saitta@humanitas.it (C.S.); giuseppe.garofano@humanitas.it (G.G.); giovanni.lughezzani@humanitas.it (G.L.); vittorio.fasulo@humanitas.it (V.F.); pietro.diana@humanitas.it (P.D.); alessandro.uleri@humanitas.it (A.U.); andrea.piccolini@humanitas.it (A.P.); stefano.mancon@humanitas.it (S.M.); paola.arena@humanitas.it (P.A.); federica.sordelli@humanitas.it (F.S.); matilde.mantovani@st.hunimed.eu (M.M.); pierpaolo.avolio@humanitas.it (P.P.A.); edoardo.beatrici@humanitas.it (E.B.); nicolo.buffi@humanitas.it (N.M.B.); 2 Department of Urology, IRCCS Humanitas Research Hospital, 20089 Rozzano, Italy; rodolfo.hurle@humanitas.it (R.F.H.); massimo.lazzeri@humanitas.it (M.L.); alberto.saita@humanitas.it (A.S.); paolo.casale@humanitas.it (P.C.); 3 Department of Urology, UC San Diego Health System, San Diego, CA 92037, USA; mfmeaghe@health.ucsd.edu (M.F.M.); kitlyuen@gmail.com (K.L.Y.); iderweesh@gmail.com (I.H.D.)

**Keywords:** age, CKD-S, functional decline, estimated glomerular filtration rate, nephrectomy, renal cell carcinoma, robotic surgery, survival analysis

## Abstract

*Background and Objectives*: to investigate the impact of age on renal function deterioration after robotic-assisted partial nephrectomy (RAPN) focusing on a decline to moderate and severe forms of chronic kidney disease (CKD). *Materials and Methods*: This is a single center prospective analysis of patients who underwent RAPN. The outcomes include the development of de novo CKD-S 3a [estimated glomerular filtration rate (eGFR) < 60 mL/min/1.73 m^2^)] and de novo CKD-S 3b (eGFR < 45 mL/min/1.73/m^2^). Multivariable analysis (MVA) via Cox regression identified predictors for CKD-S 3a/b. Kaplan –Meier Analyses (KMA) were fitted for survival assessment. Multivariable linear regression was utilized to identify the predictors of last-eGFR. *Results*: Overall, 258 patients were analyzed [low age (<50) *n* = 40 (15.5%); intermediate age (50–70) *n* = 164 (63.5%); high age (>70) *n* = 54 (20.9%)] with a median follow-up of 31 (IQR 20–42) months. MVA revealed an increasing RENAL score [Hazard Ratio (HR) 1.32, *p* = 0.009], age 50–70 (HR 6.21, *p* = 0.01), age ≥ 70 (HR 10.81, *p* = 0.001), increasing BMI (HR 1.11, *p* < 0.001) and preoperative CKD 2 (HR 2.43, *p* = 0.014) are independent risk factors associated with an increased risk of CKD-S 3a; conversely, post-surgical acute kidney injury was not (*p* = 0.83). MVA for CKD-S 3b revealed an increasing RENAL score (HR 1.51, *p* = 0.013) and age ≥ 70 (HR 2.73, *p* = 0.046) are associated with an increased risk of CKD-S 3b. Linear regression analysis revealed increasing age (Coeff. −0.76, *p* < 0.001), increasing tumor size (Coeff. −0.31, *p* = 0.03), and increasing BMI (Coeff. −0.64, *p* = 0.004) are associated with decreasing eGFR at last follow-up. We compare the survival distribution of our cohort stratified by age elderly patients experienced worsened CKD-S 3a/b disease-free survival (*p* < 0.001; *p* < 0.001, respectively). *Conclusions*: Age is independently associated with a greater risk of significant and ongoing decline in kidney function following RAPN. Recognizing the impact of aging on renal function post-surgery can guide better management practices. Further investigations are required.

## 1. Introduction

Renal cell carcinoma (RCC) constitutes 4% of global tumors, accounting for 179,368 deaths worldwide in 2020 [1,2]. Over the last decade, RCC has experienced a rise in incidence, partly due to more frequent detection using imaging technologies [3,4]. This has led to a trend of identifying cases at earlier stages [5,6]. Concurrently, partial nephrectomy (PN) has become the preferred method for managing most localized renal masses [7,8]. However, radical nephrectomy (RN) remains a cornerstone of surgical intervention, offering solid and comparable cancer outcomes to PN, with a lower risk profile for complications [9]. Conversely, while PN might result in a lesser decline in the estimated glomerular filtration rate (eGFR), a considerable number of patients may still experience a decline [9,10,11,12,13,14,15,16,17,18,19,20], and the development of an eGFR below 45 mL/min/1.73 m^2^ (CKD stage ≥ 3b) has been associated with poorer survival outcomes [21]. RCC is known to be influenced by metabolic factors [22,23,24]; therefore, exploring risk factors associated with renal functional recovery after PN is of paramount importance. While it is well-established that older age can increase mortality risks associated with renal surgery [25], the specific effects of age on the decline in kidney function following robotic-assisted partial nephrectomy (RAPN) are still debated. We sought to delve into how age affects kidney function post-RAPN, with a particular focus on the progression to moderate and severe chronic kidney disease (CKD). 

## 2. Materials and Methods

### 2.1. Study Population

This is a single center prospective study of patients who underwent RAPN for a renal mass suspicious for RCC. Approval from the Institutional Review Board was obtained. All RAPNs were executed by three experienced urologic oncology surgeons, and the decision for the type of surgery performed [radical (RN) vs. partial nephrectomy (PN)] was based on surgeons’ experience in the setting of shared decision-making. The inclusion criteria consist of patients older than 18 years affected by a localized renal mass eligible for RAPN. Patients with bilateral renal tumors, synchronous metastases, those who received neoadjuvant treatment, those with end stage renal disease, and those whose surgeries were intraoperatively converted to open surgery (*n* = 10) were excluded from the analysis. All patients underwent a preoperative three-dimensional (3D) abdominal CT scan or abdominal MRI to define the clinical stage and the anatomical characteristics of the tumor. Postoperative follow-up was conducted in accordance with the most relevant international guidelines [7,8,26].

### 2.2. Data Collected and Study Endpoints

Demographic data included age, sex, American Society of Anesthesiologists (ASA) score, Body Mass Index (BMI, kg/m^2^), presence of hypertension (HTN), diabetes mellitus (DM), clinical tumor size, pathological stage [27], RENAL score [28], warm ischemia time (WIT), operative room time (OR), and estimated blood loss (EBL). A successful surgical outcome was defined according to the Margin Ischemia Complication (MIC) index [29], defined as the presence of a negative surgical margin, WIT < 20 min, and absence of postoperative complications (Clavien Dindo > 2) [30,31]. Serum creatinine was collected preoperatively and postoperatively at 3 months, 6 months, and then annually thereafter. The CKD-EPI equation was used to calculate the estimated GFR (eGFR) [32]. Postoperative acute kidney injury (AKI) was defined as a >25% reduction in a patient’s eGFR from baseline, obtained preoperatively to discharge [33], according to the Risk/Injury/Failure/Loss/End-stage (RIFLE) criteria. The time to last creatinine was calculated from the surgery day until the most recent available creatinine measurement. The primary outcome used was the development of de novo surgically induced CKD stage 3a [estimated glomerular filtration rate (eGFR) < 60 mL/min/1.73 m^2^]. The secondary outcome was the development of postoperative eGFR < 45 mL/min/1.73 m^2^ on last follow-up (CKD-S 3b) [21,34].

### 2.3. Surgical Technique

All surgeries were performed using a transperitoneal approach. Patients were positioned at a 60-degree angle opposite to the side being operated on. An incision of 1 cm was made on the pararectal line, and access to the peritoneum was obtained according to Hasson’s technique [35]. Pneumoperitoneum was obtained by injecting CO_2_ gas until the pressure reached 14 mmHg. The setup included an 8 mm port for a 30-degree 3D video laparoscope, positioned on the outer of the border of the rectus abdominis muscle. For the robotic arms, two 8 mm trocars were inserted. In addition, two assistant ports were placed: a 12 mm port about 5 cm above the navel and a 5 mm port of the same distance below it. Following the establishment of these ports, the robotic system was docked. The next step involved mobilizing the colon and retracting it internally. Subsequent steps included dissecting the renal artery and mobilizing the kidney to reveal the tumor. The on-clamp technique was executed for all RAPNs; once the artery was clamped, a section of the renal parenchyma, approximately 0.5 cm from the tumor’s boundary, was surgically removed along with the tumor.

### 2.4. Statistical Analysis

Patients were sub-stratified by age [low age (<50); intermediate age (50–70); high age (≥70)] [25]. In our study, we first used the Kolmogorov –Smirnov test to check whether the data followed a normal distribution. For describing the data, we calculated frequencies and percentages for categorical variables. Depending on whether a quantitative variable followed a normal distribution, we either used the mean and standard deviation for analysis or the median and interquartile range (IQR) for those not normally distributed. For examining changes in continuous variables, we applied Student’s paired *t*-test for normally distributed data and the Kruskal–Wallis test for data that did not follow a normal distribution. Finally, for analyzing categorical data, we employed either Pearson’s chi-squared test or Fisher’s exact test with Yates’ correction. Multivariable analysis (MVA), via Cox regression models, was fitted to elucidate the predictors of outcomes. Kaplan–Meier analysis (KMA) was employed to evaluatively compare the survival distributions among low age, intermediate age, and high age groups for CKD-S3a, while for CKD-S3b we contrasted high age vs. intermediate and low age groups. This decision was based on the small number of events for CKD-S3b, since introducing a three-category age stratification could potentially widen the 95% confidence intervals and increase the likelihood of encountering a type 1 error. Differences between the survival distributions were evaluated using a log-rank test. Finally, a multivariable linear regression model was fitted to evaluate the predictors of eGFR at last follow-up, including an interaction term between warm ischemia time and tumor size. Data were analyzed using Stata18/SE package software (StataCorp, Houston, TX, USA), and the alpha level was set at 0.05.

## 3. Results

### 3.1. Descriptive Analysis

Overall, 258 patients were analyzed [low age *n* = 40 (15.5%); intermediate age *n* = 164 (63.5%); high age *n* = 54 (20.9%)] with a median follow-up of 31 (IQR 20–42) months. Table 1 outlines the demographics and clinical characteristics of the cohort. When sorted by age, we noted the following statistically significant different distributions. Patients older than 70 years of age had a higher proportion of males [41 (75.9%)] compared to the other groups [58 (35.4%) and 13 (24.1%), *p* < 0.001], HTN [70.39 (72.2%) vs. 82 (50.0%) and 15 (27.8%), *p* < 0.001], and DM [13 (24.1%) vs. 15 (9.1%) vs. 3 (7.5%), *p* = 0.009]. The median preoperative eGFR was lower in the over-70 age group [89.2 mL/min/1.73 m^2^ (IQR 74.7–93.7)] compared to the other groups [98.8 mL/min/1.73 m^2^ (IQR 90.3–104.4) and 109.2 mL/min/1.73 m^2^ (IQR 102.7–112.6), *p* < 0.001]. Similarly, the median postoperative eGFR showed a statistically significant decrease in the oldest age group [71.6 mL/min/1.73 m^2^ (IQR 54.1–82.4)] in contrast to the other groups [86.1 mL/min/1.73 m^2^ (IQR 72.4–99.2) and 104.1 mL/min/1.73 m^2^ (IQR 87.9–111.1), <0.001]. The last measured eGFR was significantly lower in patients older than 70 [66.5 mL/min/1.73 m^2^ (50.7–79.9) vs. 83.5 mL/min/1.73 m^2^ (66.4–94.6) vs. 99 mL/min/1.73 m^2^ (86.5–107.4), *p* < 0.001].

### 3.2. Multivariable Analysis

The MVA is depicted in Table 2a,b. MVA revealed an increasing RENAL score [Hazard Ratio (HR) 1.32, *p* = 0.009], age 50–70 (HR 6.21, *p* = 0.01), age ≥ 70 (HR 10.93, *p* = 0.001), increasing BMI (HR 1.11, *p* < 0.001), and preoperative CKD 2 (HR 2.43, *p* = 0.014) are independent risk factors associated with an increased risk of CKD-S3a; conversely, post-surgical AKI was not (*p* = 0.83). MVA for CKD-S 3b revealed an increasing RENAL score (HR 1.51, *p* = 0.013) and age ≥ 70 (HR 2.73, *p* = 0.046) are associated with an increased risk of CKD-S 3b.

### 3.3. Kaplan–Meier Analysis 

**Figure 1:**
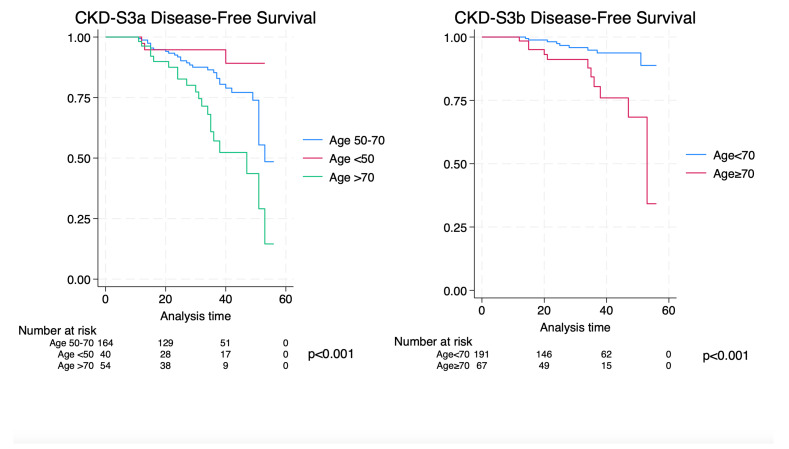
Kaplan–Meier analysis comparing age groups for CKD-S3a/b.

Figure 1 demonstrates Kaplan–Meier analyses. Comparing ages <50 vs. 50–70 vs. ≥70, 4-year CKD-S3a disease-free survival was 89.1% vs. 77.6% vs. 45.9% (*p* < 0.001); meanwhile, comparing age ≥70 vs. age < 70, 4-year CKD-S3b disease-free survival was 93.7% vs. 68.3% (*p* < 0.001). 

### 3.4. Linear Regression Model

**Figure 2:**
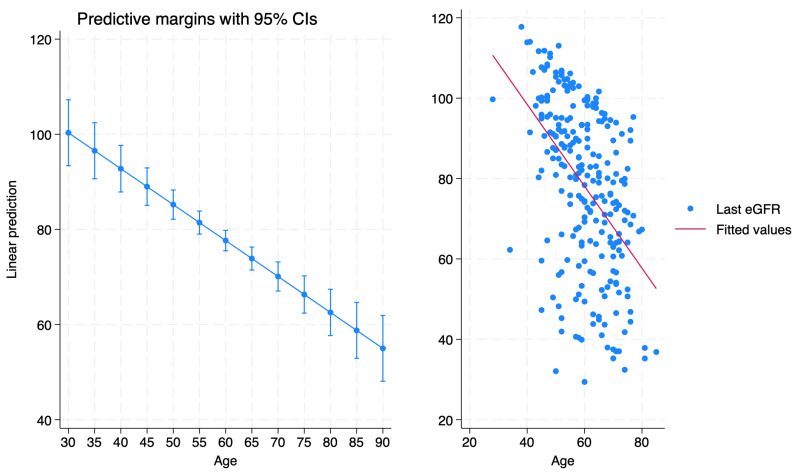
Comparison between linear prediction of eGFR and actual values across different ages. The left graph displays predictive margins with 95% confidence intervals, highlighting a decreasing trend in eGFR with increasing age. The right graph plots observe eGFR values (blue dots) against a linear regression line (red line) fitting the data, demonstrating a negative correlation between age and eGFR.

Table 3 demonstrates the linear regression model for predictors of the last eGFR. Linear regression analysis revealed the increasing age (Coeff. −0.76, *p* < 0.001), increasing tumor size (Coeff. −0.31, *p* = 0.03) and increasing BMI (Coeff. −0.64, *p* = 0.004) are associated with decreasing eGFR at last follow-up. Margins’ analysis and scatter plot are depicted in Figure 2.

## 4. Discussion

Increasing age has been advocated as a risk factor for worsened oncological and functional outcomes for RCC [25]. Nevertheless, since most of the studies amalgamate RN and PN cohorts, the impact of age on long-term renal functional outcomes after RAPN has not been well elucidated yet. We used a large prospective dataset and noted that increasing age was an independent risk factor associated with worsened functional outcomes after RAPN. We found that elderly patients were associated with a greater risk of functional decline below the threshold for CKD-S3a/b after RAPN. Furthermore, we noted an inverse linear association between increasing age and eGFR (*p* < 0.001). Cumulatively, our results suggest that increasing age is associated with renal functional decline and call consideration towards improved medical management of or a multidisciplinary approach for elderly patients scheduled for RAPN.

The previous literature suggests that age is an independent risk factor for renal functional deterioration after renal surgery. However, findings on this topic have not always been consistent, and most available studies have considered mixed cases of partial and radical renal surgery. In 2018, Martini and colleagues [36] developed a nomogram for the prediction of a significant eGFR decrease (≥25% from baseline) within 3 to 15 months post-RAPN. Another nomogram was developed in 2019 to estimate the likelihood of postoperative AKI (decrease in postoperative eGFR ≥25% from baseline) [37]. In both models, Martini et al. noted that age was associated renal functional decline (HR 1.01, *p* = 0.048; OR 1.02, *p* = 0.001, respectively). Similar results were reported by Raheem et al. [38], who retrospectively analyzed 698 T1 RCC who had undergone RAPN and noted that age (HR 1.041, *p* = 0.001) was an independent risk factor associated with an increased risk of de novo CKD. In a previous work, we reached similar findings [10]. Indeed, using a multicenter database of 1288 PN and 229 RN, we showed that age was associated with de novo eGFR < 45 (OR 1.05, *p* < 0.001). Conversely, Hamilton et al. [39] conducted a retrospective study of 1213 T1/T2 renal masses (677 PN vs. 536 RN) with CKD stage 2 and noted that age was not associated with eGFR < 45 (OR 1.0, *p* = 0.66), while RN vs. PN (OR 3.68, *p* < 0.001) and preexisting CKD [eGFR 60–45 (OR 3.30, *p* = 0.010)] were the only predictor in their multivariable logistic regression model. In this nuanced scenario of preexisting CKD, the findings of Hamilton et al. [39] highlight parenchyma preservation [13], over other clinical parameters such as age, as a strong predictor of CKD-S3b. Dissimilar to the previous studies, we used a prospective database of 258 RAPN, thus reducing the potential source of bias. Our cohort encompasses only 21 (8.1%) patients, which may have contributed to discrepancy between our findings and Hamilton et al. We noted that age was an independent risk factor associated with de novo CKD-S3a/b (HR 10.93, *p* = 0.001, HR 2.73, *p* = 0.046 respectively). Furthermore our 4-year KMA demonstrated worsened CKD-S3a/3b disease-free survival for elderly patients (*p* < 0.001; *p* < 0.001, respectively). To delve deeper into such relationships, we furthermore relied on a multivariable linear regression model and noted that increasing age was associated with eGFR decline at last follow-up (Coeff. −0.76, *p* < 0.001). Cumulatively, our findings, along with the others present in the literature, unequivocally establish age as a key risk factor for renal function decline after surgery, while, in the setting of preexisting CKD, the impact of age is marginal over the effect of parenchymal preservation [13]. Nonetheless, these findings advocate age as the main actor for renal functional recovery, thus suggesting a potential relationship between age and number of nephrons.

Indeed, renal function decline is considered a phenomenon that is age and time dependent. Denic et al. [40] explored the relationship between the number of nephrons and kidney function in 1638 individuals. The authors noted that the proportion of globally sclerotic glomeruli rose along with age, mainly due to the reduction in cortical volume. The authors concluded that a lower nephron number in healthy adults correlates with factors indicative of both a lower initial nephron endowment and a gradual loss of nephrons over time. Similar findings were reported by Konno et al. [41]. Indeed, in a population of 58 kidney donors, they showed that, in comparison to younger donors, older patients did not exhibit significant compensatory hypertrophy in the remaining kidney after surgery. However, whether the impact of age applies equally in both sexes remains controversial [42,43]. We observed a linear correlation between increasing age and a decline in estimated eGFR (*p* < 0.001), as well as between age and worsening renal function. Taken together these findings suggest that renal functional recovery after surgery is an age-related phenomenon and could advocate for more comprehensive considerations regarding conservative surgery in older patients experiencing a decline in eGFR [25]. Elderly patients should be properly counseled about the risk of achieving suboptimal functional outcomes after RAPN. Furthermore, age-specific strategies may be necessary to optimize outcomes and preserve renal function in patients undergoing surgery. In this context, elderly patients might potentially benefit from novel surgical aids and tools [44,45,46].

Renal functional decline below a threshold of 45 mL/min/1.73 m^2^ is a quality-of-care concern, since it has been associated with worsened survival outcomes [21,47]. Lane and colleagues [21] paved the way towards the concept of surgically induced CKD (CKD-S). Lane et al. [21] analyzed 4180 patients who underwent surgical procedures for suspected renal malignancies, with a median follow-up of 6.6 years. A total of 28% had a preoperative eGFR lower than 60 mL/min/1.73 m^2^, indicative of medically induced CKD (CKD-M), while 22% experienced a drop in eGFR below 60 mL/min/1.73 m^2^ only after surgery, categorizing them as having CKD-S. Their findings revealed that preoperative eGFR was a significant predictor of OS (HR 0.98, *p* < 0.001) and patients with preoperative CKD stages 3, 4, and 5 had an increased risk of death post renal surgery by 1.8, 3.5, and 4.4 times, respectively, compared to those with a normal preoperative GFR. Importantly, the postoperative eGFR only impacted survival rates in patients with CKD-M, while neither CKD-S or the postoperative eGFR significantly affected survival in patients without pre-existing, medically induced CKD. Similarly, Wu et al. [48] analyzed 4246 individuals who underwent renal surgery with a median follow-up period of 9.4 years. The authors noted that those with an eGFR < 45 mL/min/1.73 m^2^ had notably lower survival rates compared to both the no-CKD group and CKD-S patients with an eGFR between 45–60 mL/min/1.73 m^2^ (*p* < 0.001). They also found that age (HR 1.07, *p* < 0.001) was associated with an increased risk of non-renal cancer mortality. Similar results were recently reported by Nguyen et al. [25], which noted that a decline in eGFR below 45 mL/min/1.73 m^2^ was associated with an increased risk of both all-cause and non-cancer mortality. Taken together these findings underlined an interconnection between age, renal functional decline, and mortality. These findings may influence the decision-making process regarding the type of surgery (PN vs. RN) performed for patients at risk of developing CKD-S and spur considerations towards greater clinical and medical scrutiny in elderly patients.

In light of these findings, there is a growing need to tailor treatment strategies to individual patients, considering factors such as age, underlying comorbidities, and the stage of the tumor at diagnosis [49]. Ristau et. al [50] presented findings from the US National Cancer Database that highlight a diminishing benefit in overall survival linked to nephron-sparing surgery for larger renal cell carcinoma tumors (stages T1b-T2), especially in patients aged 75 and above. Here, no significant survival advantage was seen when comparing partial nephrectomy to radical nephrectomy. The study also revealed a less-pronounced benefit in larger tumors (HR 0.88; 95% confidence interval [CI], 0.83–0.94) compared to smaller tumors (T1a: HR, 0.73; 95% CI, 0.70–0.75), indicating a reduced influence of non-cancer-related factors on overall survival in advanced-stage tumors. Additionally, recent findings from multicenter research across various leading centers and comprehensive meta-analyses have indicated that, in cases of T1b and T2 renal cell carcinomas, partial nephrectomy might offer comparable oncological outcomes (for T2 disease: HR 0.71; *p* = 0.51) with added functional advantages over radical nephrectomy, particularly a lower risk of postoperative chronic kidney disease (odds ratio [OR] 0.36; *p* < 0.001). However, this functional benefit appears to decrease with more complex tumors and is associated with a higher likelihood of postoperative complications (OR, 1.74; *p* < 0.001) [9]. Our findings demonstrating worsened CKD-S3a/b disease-free survival may align with previous studies. Adding to this, it is important to consider that the lack of a significant survival advantage in patients over 75 undergoing PN vs. RN may be attributed to pre-existing diminished renal function in this age group. Furthermore, the potential benefits of nephron-sparing surgery in preserving kidney function could be offset by an increased risk of complications in these older patients. Therefore, the decision-making process for treatment strategies in elderly patients needs to carefully weigh the potential functional benefits against the likelihood of postoperative complications. In this more nuanced scenario, in elderly patients with complex renal masses, a nephron-sparing approach could be avoided. 

In our research, which is both informative and comprehensive, we must acknowledge certain limitations that could affect the interpretation and generalizability of our findings. Our study primarily focuses on the outcomes of older patients who underwent surgery at a large, tertiary referral center. This specific setting may limit the applicability of our results to smaller, less specialized centers, where patient demographics and clinical practices might differ significantly. The surgeries in our study were conducted by three skilled urologic oncology surgeons. Despite their expertise, it is important to consider the possibility of variability in surgical techniques and decisions between these operators. Such inter-operator differences, although subtle, could potentially influence patient outcomes. Another important aspect of our study is its prospective design, which generally strengthens the validity of the findings. However, we must acknowledge that our strict surgical criteria for elderly patients, particularly those with multiple comorbidities, might introduce a selection bias. This bias could exclude less resilient patients or those with more complex health issues, who may not qualify for surgery, thereby potentially skewing our results. While we have made efforts to control for a range of relevant clinical variables, such as BMI, MIC, postoperative AKI, HTN, DM, and preoperative CKD, it is important to recognize that our study could not completely eliminate all potential confounding factors. While our Kaplan–Meier analysis provides valuable insights into survival probabilities up to 48 months, we acknowledge that the median follow-up time of 33 months could be considered a limitation. This shorter median follow-up period means that our analysis may not fully capture long-term outcomes beyond this timeframe. We suggest that future studies with a longer follow-up period could provide additional insights into the long-term effects and survival probabilities associated with treatment and disease progression. Our analysis was conducted within a cohort of patients undergoing RAPN without a direct control group for comparison. This means that our findings are most pertinent to patients who are considering or are candidates for this specific surgical intervention. A direct comparison with a cohort undergoing radical nephrectomy would be valuable for a more comprehensive understanding, but our current database does not possess the necessary granularity for such an analysis. In conclusion, despite the limitations mentioned, our study offers valuable insights into the decline in renal function post-RAPN in elderly patients. These findings contribute to the growing body of evidence in urologic oncology and provide a basis for further research in this area, especially in terms of patient selection and surgical decision-making for older patients.

## 5. Conclusions

Age is independently associated with a greater risk of significant and ongoing decline in kidney function following robotic-assisted partial nephrectomy. Recognizing the impact of aging on renal function post-surgery can guide better management practices. External validation within a larger and more heterogenous cohort should be performed.

## Figures and Tables

**Table 1:** Demographics and clinical characteristics.

	Total	Age < 50	Age 50–70	Age > 70	*p*-Value
	*n* = 258	*n* = 40	*n* = 164	*n* = 54	
Sex					0.017 *
Female	92 (35.7%)	21 (52.5%)	58 (35.4%)	13 (24.1%)	
Male	166 (64.3%)	19 (47.5%)	106 (64.6%)	41 (75.9%)	
BMI median (Kg/m^2^, IQR)	25.9 (23.4–29.0)	26.1 (22.6–31.4)	25.7 (23.4–28.7)	26.2 (24.2–27.7)	0.67 ^§^
Hypertension *n* (%)					<0.001 *
No	127 (49.2%)	30 (75.0%)	82 (50.0%)	15 (27.8%)	
Yes	131 (50.8%)	10 (25.0%)	82 (50.0%)	39 (72.2%)	
Diabetes mellitus *n* (%)					0.009 *
No	227 (88.0%)	37 (92.5%)	149 (90.9%)	41 (75.9%)	
Yes	31 (12.0%)	3 (7.5%)	15 (9.1%)	13 (24.1%)	
ASA score *n* (%)					0.10 *
1	191 (74.0%)	32 (80.0%)	124 (75.6%)	35 (64.8%)	
2	32 (12.4%)	3 (7.5%)	23 (14.0%)	6 (11.1%)	
3	35 (13.6%)	5 (12.5%)	17 (10.4%)	13 (24.1%)	
Tumor size (mm)	30.0 (23.0–43.0)	30.0 (20.0–42.5)	30.0 (21.5–41.0)	35.5 (25.0–46.0)	0.17 ^§^
RENAL score *n* (%)					0.82 *
4	25 (9.7%)	4 (10.0%)	16 (9.8%)	5 (9.3%)	
5	31 (12.0%)	3 (7.5%)	21 (12.8%)	7 (13.0%)	
6	45 (17.4%)	8 (20.0%)	28 (17.1%)	9 (16.7%)	
7	52 (20.2%)	4 (10.0%)	37 (22.6%)	11 (20.4%)	
8	58 (22.5%)	11 (27.5%)	35 (21.3%)	12 (22.2%)	
9	31 (12.0%)	6 (15.0%)	16 (9.8%)	9 (16.7%)	
10	13 (5.0%)	4 (10.0%)	8 (4.9%)	1 (1.9%)	
11	2 (0.8%)	0 (0.0%)	2 (1.2%)	0 (0.0%)	
12	1 (0.4%)	0 (0.0%)	1 (0.6%)	0 (0.0%)	
Preoperative eGFR (mL/min/1.73 m^2^, IQR)	97.5 (89.1–104.8)	109.2 (102.7–112.6)	98.8 (90.3–104.4)	89.2 (74.7–93.7)	**<0.001 ^§^**
Preoperative CKD 2 *n* (%)					0.017 *
No	238 (92.2%)	39 (97.5%)	154 (93.9%)	45 (83.3%)	
Yes	20 (7.8%)	1 (2.5%)	10 (6.1%)	9 (16.7%)	
Postoperative eGFR (mL/min/1.73 m^2^, IQR)	85.09847 (69.46455–99.56562)	104.0546 (87.86871–110.9829)	86.07332 (72.39315–99.18401)	71.62138 (54.06985–82.43972)	**<0.001 ^§^**
Post-surgical AKI *n* (%)					0.14 *
No	211 (81.8%)	36 (90.0%)	135 (82.3%)	40 (74.1%)	
Yes	47 (18.2%)	4 (10.0%)	29 (17.7%)	14 (25.9%)	
eGFR at last follow-up (mL/min/1.73 m^2^, IQR)	81.6 (64.1–94.6)	99 (86.5–107.4)	83.5 (66.4–94.6)	66.5 (50.7–79.9)	**<0.001 ^§^**
Delta eGFR	9.6 (2.3–18.7)	6.6 (0.0–14.0)	9.8 (2.6–18.6)	12.7 (3.4–21.5)	0.097 *
CKD-S3a *n* (%)					**0.001** *
No	204 (79.1%)	37 (92.5%)	133 (81.7%)	34 (63%)	
Yes	54 (19.8%)	3 (7.5%)	31 (18.9%)	20 (37%)	
CKD-S3b *n* (%)					**0.003** *
No	237 (91.9%)	40 (100.0%)	153 (93.3%)	44 (81.5%)	
Yes	21 (8.1%)	0 (0.0%)	11 (6.7%)	10 (18.5%)	
Length of follow-up (months, IQR)	33.0 (20.0–42.0)	35.5 (16.5–44.5)	33.0 (21.0–42.0)	31.0 (16.0–37.0)	0.21 *
OR time (min, IQR)	135.5 (113.0–168.0)	125.0 (109.5–146.5)	140.0 (112.0–169.0)	148.0 (116.0–173.0)	0.13 ^§^
WIT (min, IQR)	12.0 (10.0–16.0)	12.0 (9.0–17.0)	12.0 (10.0–17.0)	12.0 (9.0–16.0)	0.65 ^§^
EBL (ml, IQR)	70.0 (40.0–100.0)	70.0 (37.5–100.0)	70.0 (40.0–100.0)	75.0 (30.0–200.0)	0.92 ^§^
Surgical margin *n* (%)					0.47 *
Negative	249 (96.5%)	38 (95.0%)	160 (97.6%)	51 (94.4%)	
Positive	9 (3.5%)	2 (5.0%)	4 (2.4%)	3 (5.6%)	
MIC *n* (%)					0.36 *
No	46 (17.8%)	9 (22.5%)	25 (15.2%)	12 (22.2%)	
Yes	212 (82.2%)	31 (77.5%)	139 (84.8%)	42 (77.8%)	

Abbreviations: ^§^ Kruskal–Wallis test; * Pearson’s chi-squared test; BMI: Body Mass Index; ASA: American Society of Anesthesiologists; eGFR: estimated glomerular filtration rate; CKD: chronic kidney disease; AKI: Acute Kidneys Injury; OR time: operative room time; WIT: warm ischemia time; MIC: Margin Ischemia Complication. Parameters for which a statistically significant difference is present are in bold.

**Table 2:** (**a**) Multivariable analysis for predictors of eGFR CKD-S3a; (**b**) Multivariable analysis for predictors of eGFR CKD-S3b.

**(a)**				
**Covariates**	**HR**	**[95% conf. Interval]**		***p*-Value**
RENAL Score (Continuous)	1.32	1.07	1.62	0.009
Tumor Size (Continuous)	1.00	0.98	1.02	0.924
Age	Reference < 50			
50–70	6.21	1.55	24.88	0.01
≥70	10.93	2.64	45.30	0.001
BMI (Continuous)	1.11	1.05	1.18	<0.001
MIC (Yes vs. No)	1.25	0.59	2.64	0.56
Post-Surgical AKI (Yes vs. No)	1.08	0.55	2.12	0.83
HTN (Yes vs. No)	1.12	0.56	2.23	0.748
DM (Yes vs. No)	1.83	0.86	3.91	0.116
Preoperative CKD 2a (Yes vs. No)	2.43	1.20	4.93	0.014
(**b**)				
**Covariates**	**HR**	**[95% conf. Interval]**		***p* > |z|**
RENAL Score (Continuous)	1.51	1.09	2.10	0.013
Tumor Size (Continuous)	1.02	0.99	1.05	0.192
Age ≥70 (Yes vs. No)	2.73	1.02	7.32	0.046
BMI (Continuous)	1.08	0.98	1.18	0.116
MIC (Yes vs. No)	0.64	0.24	1.66	0.354
Post-Surgical AKI (Yes vs. No)	0.69	0.24	1.94	0.476
HTN (Yes vs. No)	3.77	0.76	18.60	0.103
DM (Yes vs. No)	2.06	0.68	6.27	0.203
Preoperative CKD 2a (Yes vs. No)	1.58	0.51	4.93	0.43

Abbreviations: BMI: Body Mass Index; AKI: Acute Kidneys Injury; HTN: Hypertension; DM: Diabetes Mellitus; CKD: chronic kidney disease.

**Table 3:** Linear regression model.

Covariates	Coefficient	[95% conf. Interval]		*p*-Value
Age (Continuous)	−0.76	−0.98	−0.54	<0.001
Tumor Size (Continuous)	−0.31	−0.58	−0.03	0.03
BMI (Continuous)	−0.64	−1.07	−0.20	0.004
WIT (Continuous)	0.03	−0.88	0.93	0.952
Post-Surgical AKI (Yes vs. No)	−4.84	−10.54	0.87	0.096
Interaction WIT-Tumor Size	0.01	−0.01	0.04	0.19

Abbreviations: BMI: Body Mass Index; AKI: Acute Kidneys Injury; OR time: operative room time; WIT: warm ischemia time.

## Data Availability

Data are contained within the article.
